# Investigating the role of executive attentional control to self-harm in a non-clinical cohort with borderline personality features

**DOI:** 10.3389/fnbeh.2014.00274

**Published:** 2014-08-20

**Authors:** Jennifer Drabble, David P. Bowles, Lynne Ann Barker

**Affiliations:** Brain, Behavior and Cognition Group, Faculty of Development and Society, Sheffield Hallam University, Sheffield, UK

**Keywords:** executive control, attention, self-harm, borderline personality disorder

## Abstract

Self-injurious behavior (or self-harm) is a frequently reported maladaptive behavior in the general population and a key feature of borderline personality disorder (BPD). Poor affect regulation is strongly linked to a propensity to self-harm, is a core component of BPD, and is linked with reduced attentional control abilities. The idea that attentional control difficulties may provide a link between BPD, negative affect and self-harm has yet to be established, however. The present study explored the putative relationship between levels of BPD features, three aspects of attentional/executive control, affect, and self-harm history in a sample of 340 non-clinical participants recruited online from self-harm forums and social networking sites. Analyses showed that self-reported levels of BPD features and attentional focusing predicted self-harm incidence, and high attentional focusing increased the likelihood of a prior self-harm history in those with high BPD features. Ability to shift attention was associated with a reduced likelihood of self-harm, suggesting that good attentional switching ability may provide a protective buffer against self-harm behavior for some individuals. These attentional control differences mediated the association between negative affect and self-harm, but the relationship between BPD and self-harm appears independent.

## Introduction

Self-harm, intentional injuring of one’s body tissue, is a core feature of Borderline Personality Disorder (BPD) and may be also seen in a diverse range of psychiatric disorders (Briere and Gil, 1998). Self-harm is thought to have a general population prevalence of around 4%, rising to 21% in clinical populations (Briere and Gil, 1998), and 89% in individuals diagnosed with BPD (Zanarini et al., 2008). Estimates show that there are 140,000–170,000 admittances to UK hospitals for self-inflicted injury per year (Hawton et al., 2007), and self-harm constitutes one of the commonest reasons for hospital admission (Weston, 2003). While the exact role of self-harm to the maintenance or attempted management of psychiatric symptoms remains to be established, it may represent a maladaptive form of affect regulation (see Klonsky, 2009, for a review).

Self-harm comprises one of several key diagnostic criteria for BPD together with frantic efforts to avoid real or imagined abandonment, unstable interpersonal relationships, impulsivity, suicidality, identity disturbance and marked inappropriate anger (Lieb et al., 2004; American Psychiatric Association, 2013). BPD affects between 1.2–6% of the general population (Crowell et al., 2009), and around 10–20% of psychiatric populations, a relatively large proportion of total number of individuals referred to psychiatric services (Lieb et al., 2004). The elevated risk for individuals with BPD to be admitted to hospitalization for self-harm is considerable: Sansone et al. (2005) found that BPD patients reported more than twice the number of self-harm behaviors than patients diagnosed with another psychiatric disorder. However despite prevalence of mutilative acts and high risk of suicidality in BPD patients, self-harming behaviors need not be present to merit a diagnosis of BPD. It is likely that propensity for self-harm in BPD is a poor prognostic indicator compared to BPD patients who do not self-harm, as BPD patients with self-harm tend to be significantly more symptomatic, prone to suicide ideation, and have more recent suicide attempts than those BPD patients without self-harm (Dulit et al., 1994; Soloff et al., 1994).

Prevalence and frequency of self-injurious behavior in normal and psychopathological groups suggest that some individuals may engage in self-harm to serve some adaptive function, at least in the short-term. This behavior may be “adaptive” insofar as it operates as an anti-dissociation mechanism that re-affirms an individual’s desire to feel (Klonsky, 2007; American Psychiatric Association, 2013). Additionally, self-harm may serve as a means to elicit a response from others and avoid abandonment. However, the most frequently reported reason for engaging in self-harm in chronic BPD patients (Brown et al., 2002) and non-clinical BPD samples (Gratz and Roemer, 2008; Klonsky, 2009) is relief of negative emotion. Hence, for some individuals self-harm appears to be a way of self-soothing and coping with stress and negative affect (Gallop, 2002). Of course, chronic self-harm is a dangerous method of emotion regulation (Mikolajczak et al., 2009), and there is increased likelihood of suicide in self-harmers compared to non-self-harmers in the general population (Hawton et al., 2003; Hawton and Harriss, 2007). Additionally, research shows that self-harmers have significantly worse physical and social functioning and reduced quality of life compared to non-self-harmers in the general population (Sinclair et al., 2010).

Despite the prevalence of self-harm in individuals with BPD and in the general population, and the subsequent burden on health care services, it is surprising that potential pathways to self-harm behavior are not well understood (Glassman et al., 2007). One possibility is that reduced executive function ability may underlie self-harm by diminishing the capacity to self-regulate (LeGris and Van Reekum, 2006). Executive function(s) refers to a range of metacognitive capacities (higher-order attentional and control processes) that co-ordinate/maintain, initiate or inhibit other cognitive and emotional processes (Miyake et al., 2000; Barker et al., 2010; Morton and Barker, 2010) and govern self-ordered, context-appropriate and goal-directed activity (Baddeley and Wilson, 1988; Burgess and Shallice, 1996; Burgess, 2003; Strauss et al., 2006). Several theories posit a central role of attention to executive function (Stuss and Alexander, 2000; Anderson, 2003; Giesbrecht et al., 2004; Jurado and Roselli, 2007; Muscara et al., 2008; Daches et al., 2010; Spada et al., 2010). Derryberry and Reed (2002) defined attentional control as comprising three factors: (a) ability to focus and sustain attention; (b) ability to shift attention from one task to another requiring inhibition of response contingencies to the first task in order to engage with the second task; and (c) flexible thought generation.

The notion that impaired executive/attentional control processes might mediate self-harm in BPD individuals deserves further investigation because key symptom clusters characterizing the disorder indicate poor behavioral regulation (Coolidge et al., 2004), an important marker of executive dysfunction in other patient groups (Morton and Barker, 2010). Affective instability indicated by inappropriate anger, impulsivity and risk-taking behavior are core features of BPD, and are also seen, to a lesser or greater degree, in neuropathological groups with executive dysfunction (Barker et al., 2010, 2011). Diminished inhibitory capacity increases the likelihood that individuals act on dominant and potentially maladaptive tendencies; in the case of individuals with BPD this may be self-harm. However, that said the precise executive processes diminished in BPD individuals remains to be established, although evidence suggests that they may generally comprise diminished attentional control.

LeGris and Van Reekum (2006) conducted a meta-analysis and found that 86% of studies reviewed confirmed some degree of executive function impairment in BPD individuals; the deficits most often reported fell within the category of attentional impairment. Ayduk et al. (2008) investigated the relationship between attentional control, rejection sensitivity and BPD features in a non-clinical sample using the Attentional Control Scale (Derryberry and Reed, 2002). Results showed that the association between BPD features and level of rejection sensitivity was attenuated in individuals with good attentional control. This finding suggests that good attentional control may provide some emotional buffer to override prepotent maladaptive thought patterns and inhibit dominant and maladaptive behavioral patterns in the face of perceived rejection/abandonment.

In other work Posner and Petersen (1990) defined attentional control as comprising three different but interrelated functions; alerting (achieving and maintaining an alert state), orientating, and executive control (conflict resolution/inhibition). Although the Posner and Petersen (1990) model is somewhat conceptually distinct from Derryberry and Reed (2002) model of attentional control, both share some definitional overlap and correspond well with Miyake et al. (2000) categorization of executive functions. Importantly, executive control, including orientating to, switching, focussing, and/or inhibiting attention and other cognitive processes, is integral to each theory (Posner and Petersen, 1990; Derryberry and Reed, 2002; Miyake et al., 2000).

To summarize, there is evidence to suggest that individuals with BPD have diminished executive functions; specifically they seem to exhibit deficits in attentional control and inhibiting maladaptive thoughts and behaviors. It has been suggested that they may self-harm in order to compensate for diminished affective/executive control, thus providing an outlet for emotional distress that cannot be regulated by normal cognitive and affective regulatory processes. However, less is known about what functions might contribute to self-harm in non-clinical groups with and without BPD features.

The present study investigated whether components of attentional control (shifting, focusing and flexibility) as measured by the Attentional Control Scale (ACS; Derryberry and Reed, 2002) along with BPD features would be associated with self-harm likelihood in a non-clinical sample. We predicted that deficits in specific components of attentional control (focusing, shifting, and flexibility) would be related to BPD features and self-harm. We also anticipated that attentional control would moderate the association between BPD features and self-harm.

## Materials and methods

### Participants

A self-referring non-clinical sample (*N* = 340) of participants was recruited via advertisements placed on general social networking sites such as Facebook and Twitter, and in topic-relevant forums such as the “self-harm awareness group”.^1^ Participants were aged 16–62 (*M* = 26.94, *SD* = 10.14), and 279 (82%) were women; 117 (34.41%) participants reported previous self-harm. The two groups did not differ significantly by gender (*X*^2^ (1, *N* = 340) = 0.35, *p* > 0.05), but there were significant age differences (*U* = 9251.00, *Z* = −4.41, *p* < 0.001); participants who reported prior self-harm were significantly younger. This corresponds to the pattern of diminished BPD symptoms with advancing age shown in the literature and clinical populations (Zanarini et al., 2007).

### Materials and procedure

All measures were completed online via SurveyMonkey. The current research project was approved by the University’s Research Ethics Committee. Informed consent was obtained via an information screen containing details of the study, issues of confidentiality and the right to withdraw. Potential participants recruited to the study progressed beyond the initial consent screen to provide gender and age details before completing the self-report measures described below.

#### Attentional control measure

##### The Attentional Control Scale (ACS; Derryberry and Reed, 2002).

The ACS is a 20-item, self-report measure of attentional control. Participants respond to a four-point response scale (almost never, sometimes, often, always). High scores on the ACS represent good capacity to voluntarily control attention, whereas low scores are associated with attentional rigidity. A psychometric analysis of the scale by Fajwowska and Derryberry (2010) suggest that the ACS has three subscales; the attention focusing subscale has nine items and refers to the ability to focus and maintain attention (example item: “It’s very hard for me to concentrate on a difficult task when there are noises around”). The attention shifting subscale has six items and refers to the ability to shift attention between focal points (example item: “I can quickly shift from one task to another”). The flexibility/divided attention subscale has five items (example item: “I have trouble carrying on two conversations at once”). In the current study, the focusing subscale demonstrated good internal consistency (*α* = 0.75), and shifting and flexibility subscale alphas demonstrated acceptable internal consistency for small scales (*α* = 0.58 and 0.56, respectively).

#### Measures of Borderline Personality features

##### Structured Clinical Interview for DSM-IV Axis II Personality Disorders Screening Questionnaire (SCID-II-SQ; First et al., 1997).

The SCID-II-SQ is a self-report screening measure used to assess broad personality disorder features. The current study used the 15 item BPD subscale (example item: “Have you often become frantic when you thought that someone you really cared about was going to leave you?”) and was modified from the original “yes/no” response option to measure symptoms dimensionally on a four-point response scale (0 = never or not at all, 1= sometimes or a little, 2 = often or moderately, 3 = very often or extreme) based on previous work with non-clinical samples (e.g., Dreessen et al., 1999; Meyer et al., 2005; Bowles and Meyer, 2008). Two self-harm related items were removed (“Have you tried to hurt or kill yourself or threatened to do so?” and “Have you ever cut, burned, or scratched yourself on purpose?”) to avoid colinearity with the measure of self-harm leaving 13 items in the scale. Internal consistency for this version of the BPD subscale has been reported as good (Cronbach’s *α* = 0.83, Meyer et al., 2005), and the 13-item version used in the present study was at least as reliable (*α* = 0.90).

##### The Short Coolidge Axis Two Inventory (SCATI; Coolidge, 2001).

The SCATI is also a self-report measure of personality disorder features. The five-item BPD scale was used (example item: “I am very afraid of being abandoned by someone”), and participants responded on a four-point scale (strongly false, more false than true, more true than false, strongly true). There is one self-harm related item on the scale (“I have repeatedly made suicidal threats or gestures, or I have repeatedly hurt myself on purpose”), which was removed prior to analyses to again avoid colinearity with the self-harm measure leaving four remaining items which demonstrated acceptable internal consistency in the current study (*α* = 0.70). The total raw scores on the Personality Assessment Inventory (PAI) can be converted to T-Scores, which are calibrated with reference to a matched community sample. Individuals with scores <60 T are considered to have fairly healthy personality organization. Scores of 60–69 T is a moderate elevation and individuals may display increasing anger and dissatisfaction. Scores of 70 T and above are considered elevated with problematic symptoms of impulsivity and interpersonal relationships. Scores greater that 90 T are generally seen only in clinical samples and suggest markedly elevated symptoms, possibly an individual in crisis.

##### The Personality Assessment Inventory (PAI; Morey, 1991).

This measure is a self-administered scale used for clinical assessment of adults. The borderline features scale (PAI-BOR) includes four subscales: affective instability, identity problems, negative relationships and self-harm. The self-harm subscale was removed from analyses, and internal consistency for the remaining 18 items was good (*α* = 0.84).

The total raw scores on the PAI can be converted to a T-Score based on normative data and uses T-scores that have a mean of 50 and a standard deviation of 10. Individuals with scores <60 T are considered to have fairly healthy personality dimensions. Scores of 60–69 T represent a moderate elevation and may indicate tendency to anger and dissatisfaction. Scores of 70 T and above are indicative of problematic symptoms in interpersonal relationships and impulsivity. Scores greater that 90 T are generally seen only in clinical samples and indicate markedly elevated symptoms, possibly an individual in crisis.

PAI-BOR T-scores for the no self-harm group ranged from 37–90 (*M* = 60.47, *SD* = 10.27) representing moderate elevation of personality traits, which is consistent with other non-clinical samples (e.g., Trull, 1995; Gardner and Qualter, 2009). In the no self-harm group, 42 participants (18.83%) had T-scores of 70 or above, which is considered to be the cut-off point that indicates presence of significant BPD features (Trull, 1995). T-Scores for the prior self-harm group ranged from 45–100 (*M* = 73.42, *SD* = 12.50), which is consistent with T-scores observed in clinical BPD samples (e.g., Jacobo et al., 2007). In the prior self-harm group, 72 participants had T-scores of 70 or above, likely reflecting problematic elevation of BPD features and indicating that individuals in non-clinical samples may show relatively high levels of borderline PD traits. T-scores differed significantly between the prior self-harm group and the no self-harm group (*U* = 5602, *Z* = −8.65, *p* < 0.001).

#### Measure of affect

##### The Positive and Negative Affect Schedule (PANAS; Watson et al., 1988).

The PANAS consists of two 10-item scales measuring positive (e.g., “enthusiastic”, “proud”) and negative (e.g., “irritable”, “nervous”) affect. Participants rate to what extent they generally experience each item on a five-point response scale ranging from “not at all” to “extremely”. Data from the current study showed high internal consistency for negative and positive scales (both *α*s = 0.92).

#### Self-harm measure

##### Deliberate Self-Harm Inventory (DSHI; Gratz, 2001).

The DSHI is a 17-item self-report questionnaire developed to measure frequency, severity and type of self-harming behavior. Participants’ rate how often they have intentionally engaged in each of the 17 behaviors (e.g., “Have you ever intentionally, on purpose, cut your wrist, arms, or other areas of your body without intending to kill yourself? If yes, how many times have you done this?”). Following completion of the measures, participants were encouraged to comment on their participation in the study (e.g., “Do you have anything you would like to add that was not asked about in this questionnaire?”) A number of participants reported they had difficulty estimating the number of times they had engaged in each of the behaviors, therefore using total number of self-harm injuries as a variable proved to be problematic. Consequently, we used the DSHI to distinguish between participants who self-harmed and those who did not.

## Results

Items relating to self-harm behaviors were removed from BPD scales to avoid colinearity with the outcome measure. Given that the three BPD scales were measuring the same underlying construct, and the correlations between the measures were moderate to large in magnitude (*r*s = 0.56−0.84), we created a composite variable representing BPD features, in order to provide more reliable measures (e.g., Cheavens et al., 2005; Sprague and Verona, 2010). Individual scores were standardized (*Z*-transformed) and then summed in order to create an overall index of BPD features. This standardized BPD scale with the self-harm related items removed demonstrated good internal consistency (35 items, *α* = 0.93).

Table 1 shows descriptive data for measures used in the current study by self-harm group (prior self-harm vs. no self-harm). Individuals who reported previous self-harm had significantly higher scores on BPD features and negative affect, and significantly lower scores on positive affect, shifting, and flexibility compared to the non-self-harm group.

**Table 1:** **Descriptive statistics for mood, BPD features and attentional subscales**.

**Measure**		**Prior self-harm (*n* = 117)**	**No self-harm (*n* = 223)**	
	**Min-Max**	**Mean (SD)**		
Negative affect	10–49	28.79 (8.79)	24.53 (9.25)	*t*_(338)_ = −4.11**
Positive affect	10–48	26.17 (8.26)	29.28 (9.08)	*t*_(338)_ = 3.10*
Focusing	9–36	22.15 (4.19)	21.48 (4.68)	*t*_(338)_ = −1.31
Shifting	6–24	14.47 (2.54)	16.37 (3.16)	*t*_(338)_ = 5.61**
Flexibility	5–20	11.17 (2.24)	11.84 (2.93)	*t*_(338)_ = 2.17*
Combined Borderline Scale (*Z*-scores)	−6.78–6.34	0.51 (0.83)	−0.33 (0.76)	*t*_(338)_ = −9.39**

* p < 0.05; ** p < 0.001.

Table 2 shows correlations between the scales used in the current study. Results of Pearson’s correlational analyses showed that ACS subscales were generally weakly to moderately correlated, indicating that ACS subscales indexed some shared processes. Negative affect scores correlated with BPD features, and negatively correlated with all three of the ACS subscale scores in the prior self-harm group, whereas it was positively correlated with focusing and shifting in the no self-harm group. The flexibility subscale scores of the ACS correlated negatively with BPD feature scores, suggesting low flexibility ability in the presence of BPD features. BPD features scores also correlated with negative affect, and inversely with positive affect.

**Table 2:** **Correlations between measurement scale scores**.

	**(1)**	**(2)**	**(3)**	**(4)**	**(5)**
(1) Negative affect	–				
(2) Positive affect	−0.24**	–			
(3) ACS—Focusing	−0.14*	0.19**	–		
(4) ACS—Shifting	−0.10	0.22**	0.48**	–	
(5) ACS—Flexibility	−0.20**	0.36**	0.32**	0.35**	–
(6) BPD features	0.71**	−0.42**	−0.07	−0.10	−0.23**

*p < 0.05; ** p < 0.001. Key: ACS = Attentional control scale; BPD features = Combined borderline scales.

A hierarchical logistic regression model was used to examine possible contribution of affect, BPD features, and attentional control to the probability of reporting previous episodes of self-harm (see Table 3). Self-harm was, therefore, the criterion variable. We decided that a binary variable simply indicating whether individuals had ever engaged in self-harm was most appropriate (see Section Materials and Methods). The variable was coded 1 to indicate prior self-harm and 0 to indicate no prior self-harm. Affect (as measured by the PANAS) was entered in the first block due to the important role negative affect plays in self-harm behavior. The BPD variable was entered in the second step to examine whether BPD features predicted self-harm likelihood separately from affect. The attentional control variables (focusing, shifting, and flexibility) as measured by the ACS were entered in the final step of the regression to examine whether deficits in specific components of attentional control would partially explain the association between BPD and self-harm.

**Table 3:** **Hierarchical Logistic Regression testing main effects of affect; attentional control; and BPD features on prior incidence of self-harm**.

	***R*^2^**	**Odds ratio**	**95% CI**
	**$R_{a}^{2}-R_{b}^{2}$**		***Lower–Upper***
**Step 1**	0.06–0.08		
Negative affect		1.05*	1.02–1.07
Positive affect		0.97*	0.94–0.99
**Step 2**	0.22–0.31		
Negative affect		0.94*	0.91–0.98
Positive affect		1.01	0.98–1.04
BPD		5.99**	3.64–9.88
**Step 3**	0.34–0.47		
Negative affect		0.94*	0.90–0.99
Positive affect		1.02	0.99–1.06
BPD		7.51**	4.26–13.25
Focusing		1.20**	1.11–1.30
Shifting		0.66**	0.58–0.75
Flexibility		1.04	0.93–1.18

Note: $R_{a}^{2}$ = Cox and Snell, $R_{b}^{2}$ = Nagelkerke, * p < 0.05; ** p < 0.001.

The full model containing all predictors was significant (χ^2^(5) = 140.79, *p* < 0.001) compared to the constant only model, indicating that the full model distinguished between participants who reported instances of self-harm and those who did not. The model as a whole explained between 34% (Cox and Snell *R*^2^) to 47% (Nagelkerke *R*^2^) of the variation in self-harm and correctly classified 80.60% of cases. There were six independent variables at the final step, four of which made a unique and significant contribution to the probability of reporting self-harm. (see Table 3).

In the final step of the regression odds ratios indicated that BPD features most strongly predicted likelihood of self-harm, and no mediating effects of the added attentional control variables were indicated. Focusing and shifting variables were associated with prior self-harm likelihood. Higher shifting scores were associated with lower rates of self-harm, and focusing appeared to have a positive association with self-harm. These associations were independent of BPD and raise the possibility that they may interact with BPD features in their association with self-harm. The positive association between self-harm and focusing is in the opposite direction to that shown in simple *t*-tests (Table 1), and may suggest a suppressor effect of either affect or BPD that is only apparent when analyzed together in a regression. Alternatively, there may be an interactive effect of BPD and focusing, and this, along with a similar interaction between shifting and BPD was explored.

To do this, interaction terms were created as the products of standardized (*Z*-transformed) versions of the BPD variable and the focusing and shifting variables. The interactive effects of BPD and focusing ability and of BPD and shifting ability were tested in two separate hierarchical logistic regressions. In each regression the two predictor variables were entered in the first step, and the interaction term was entered into the second. In both cases the interaction terms were uniquely significant (see Tables 4, 5).

**Table 4:** **Hierarchical Logistic Regression testing interaction effects of BPD features and focusing on prior incidence of self-harm**.

	***R*^2^**	**Odds ratio**	**95% CI**
	**$R_{a}^{2}-R_{b}^{2}$**		***Lower–Upper***
**Step 1**	0.21–0.29		
BPD		3.85**	2.72–5.45
Focusing		1.35*	1.03–1.75
**Step 2**	0.25–0.34		
BPD		4.21**	2.86–6.20
Focusing		1.18	0.89–1.58
BPD X Focusing interaction		2.01**	1.40–2.88

Note: $R_{a}^{2}$ = Cox and Snell, $R_{b}^{2}$ = Nagelkerke, * p < 0.05; ** p < 0.001.

**Table 5:** **Hierarchical Logistic Regression testing interaction effects of BPD features and shifting on prior incidence of self-harm**.

	***R*^2^**	**Odds Ratio**	**95% CI**
	**$R_{a}^{2}-R_{b}^{2}$**		***Lower–Upper***
**Step 1**	0.26–0.37		
BPD		3.68**	2.62–5.16
Shifting		0.46**	0.34–0.62
**Step 2**	0.28–0.39		
BPD		4.63**	3.07–6.98
Shifting		0.45**	0.33–0.62
BPD X Shifting interaction	1.75*	1.19–2.59

Note: $R_{a}^{2}$ = Cox and Snell, $R_{b}^{2}$ = Nagelkerke, * p < 0.05; ** p < 0.001.

**Figure 1:**
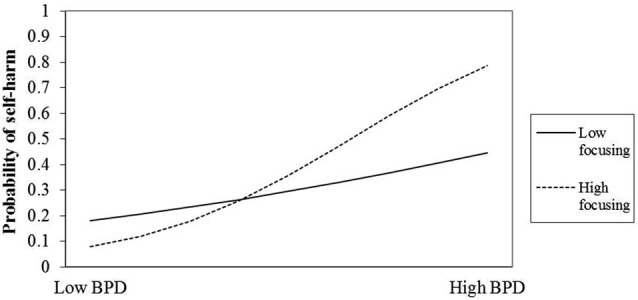
**Interaction of BPD and Focusing ability on likelihood of prior self-harm**.

**Figure 2:**
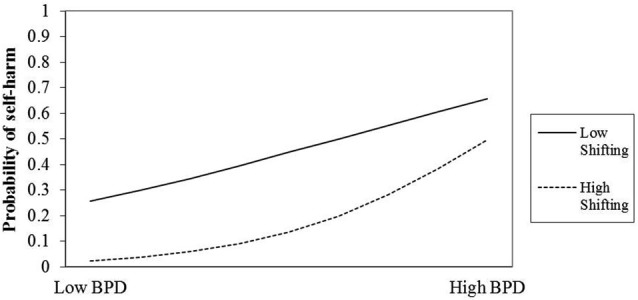
**Interaction of BPD and Shifting ability on likelihood of prior self-harm**.

Plots were created to help interpret the interactions (see Figures 1, 2). The plots indicate that those two attentional control factors differentially moderated the association between BPD and rates of self-harm. For individuals low in BPD, high focusing ability appears to reduce the risk of self-harm, yet increase the risk for those high in BPD features. One possibility is that focusing is a protective factor for some, and a rumination-like risk factor for others.

The picture with shifting ability is somewhat different. The plot suggests that for those with pronounced BPD features shifting ability has little bearing on self-harm risk. However, among those individuals with few BPD features, reduced shifting ability may pose a slightly elevated self-harm risk.

## Discussion

The current study investigated the relationship between BPD features, three aspects of attentional/executive control (shifting, focusing and flexibility), affect, and self-harm in a large non-clinical sample. The hierarchical logistic regression showed that BPD ratings and attentional focusing predicted self-harm incidence, although the pattern of data was not entirely as anticipated with high attentional focusing scores increasing the likelihood of a prior self-harm history in those rating high BPD features. The ability to shift attention was associated with a reduced likelihood of self-harm.

As hypothesized, high BPD scores were associated with greater likelihood of an individual reporting previous self-harm. Our findings demonstrate the importance that BPD features play in propensity to self-harm in a non-clinical sample. There is evidence that individuals drawn from non-clinical populations with high levels of BPD features show social and occupational problems along with impaired executive function ability compared to those with few or no BPD features (Trull et al., 1990; Fossati et al., 2004; Ayduk et al., 2008). Most research with BPD groups has centered on those with a clinical diagnosis meaning that less is known about how BPD features might drive maladaptive behavior in non-clinical groups. Our findings reproduce the strong association shown between BPD features and self-harm likelihood in clinical cases indicating that despite possible differences between clinical and non-clinical BPD there are also some shared processes that potentially transcend a BPD diagnosis in relation to self-harm. Most psychiatric disorders can be considered on a continuum from complete absence of symptoms, for example in remittance, to clinically severe (Tyrer, 2009). Our findings support the dimensional approach to psychiatric disorders and illustrate the importance of investigating functions in a range of participants who may present along the BPD spectrum.

Our results showed that high focusing ability reduced self-harm likelihood for individuals low in BPD features but increased the risk for those rating themselves highly on BPD features. Thus when high BPD features are present a good capacity to focus attention is likely directed in some maladaptive way. BPD features also correlated with negative affect: these findings raise the possibility that high focusing might manifest as ruminative perseverative thought patterns that influence behavior and affect. What is not clear is whether high focusing is targeted at potential self-harming behavior or instead functions to precipitate self-harm. The former is more plausible because self-harmers tend to report immediacy and urgency when self-harming that is then followed by catharsis. Arguably, it might be the case that high focusing ability functions to maintain some BPD features. Key features of BPD measured by our composite scale include fluid sense of self, emotional instability, feelings of and expression of rage, fear of abandonment, unstable but intense relationships and impulsivity. Thus, intenseness of relationships for example might be a consequence of over-focusing on the other, and also over-focusing on the possibility of abandonment. Likewise exaggerated anger responses might arise due to over-focusing on perceived slights or suspected indications of future abandonment. In addition, the finding that low flexibility in attentional control is associated with high BPD features supports the notion the high focusing might drive and/or maintain perseverative and anxiety inducing cognitions that ultimately lead to self-harm because the individual cannot switch attention “off topic”. High levels of focusing in people with low BPD feature ratings may protect against self-harm risk by enabling the individual to override prepotent and maladaptive thought patterns.

Present findings indicate that attentional shifting ability had little bearing on self-harm risk in those who rated themselves high on BPD features. This finding corresponds well to the notion that those high in BPD features may be highly focused upon thoughts that precipitate negative affect and self-harm. Thus, we see a pattern of relationships emerging whereby the “maintaining” function of high focusing makes most demand on capacity constrained attentional resources in those with high BPD features, at the expense of attentional flexibility and attentional switching. Our findings also show an association between low attentional shifting ability and slightly elevated self-harm risk for those individuals with few or no BPD features. Attentional shifting is not a unitary process: ability to reallocate capacity-constrained attentional resources to a different intrinsic or extrinsic stimulus depends upon inhibition of earlier focus. Thus inhibitory capacity will affect attentional shifting ability, when reduced it should make attentional switching difficult due to resource competition. In addition, emotional stimuli have been shown to be more resistant to inhibition than non-emotional stimuli (Schulz et al., 2007), and this may be particularly salient for those high in BPD features. There is also some suggestion that low inhibitory ability and high urgency may mediate rash behavior across a range of groups and disorders (Gay et al., 2008). Consequently, good attentional switching ability may provide a protective buffer against self-harm behavior for some individuals by reducing the likelihood of pathological focusing and perseverative thought patterns (Judah et al., 2014).

Individuals may self-harm for a variety of reasons including reducing negative affect and arousal, as an anti-dissociation mechanism (also referred to as “feeling generation”), as a way of avoiding suicide, reinforcing personal boundaries, as self-punishment, or as a method of sensation seeking (Klonsky, 2007). Within this framework anti-dissociation refers to capacity of self-harm to ameliorate sense of depersonalization in BPD (Klonsky, 2007; American Psychiatric Association, 2013), and is generally considered to be distinct from the graver and psychotic disconnect from reality defined as “dissociation” in other disorders such as schizophrenia and Bipolar Disorder. Although the current study did not include a specific measure of social functioning, the literature suggests self-harmers have significantly worse physical and social functioning and reduced quality of life compared to non-self-harmers in the general population (Sinclair et al., 2010). This includes a significant and persistent risk of suicide 15 years after presenting at hospital with a self-harm injury (Hawton et al., 2003). However, it is important to note that in the current study, the sample of participants likely consisted of relatively higher-functioning individuals, as participants were not recruited from mental health services or hospitals, which are typical treatment sites for lower functioning individuals with a BPD diagnosis (Sansone et al., 1998). Despite this, participants did endorse a high number of BPD features, particularly in the self-harm group. Research suggests that high BPD features (e.g., individuals who score above the clinically significant cut-off point of 70 T on the PAI-BOR) are associated with poorer outcomes such as academic difficulties, meet criteria for a mood diagnosis, and experience interpersonal dysfunction, even within a nonclinical population (Trull et al., 1997).

The development of adaptive flexible attentional control might pose a potentially useful therapeutic goal for those high in BPD features. Mindfulness refers to the practice of non-reactive attention to the present moment, focusing on thought, emotions and bodily sensations as well as environmental stimuli (sounds and smells) even if they are unwanted or unpleasant whilst accepting their impermanence (Linehan, 1993). Increased mindfulness skills appear to improve psychological functioning by cultivating an adaptive form of self-focused attention that reduces rumination and emotional avoidance, and improves behavioral self-regulation (Lynch et al., 2006; Baer, 2009; Selby et al., 2009). This may be a fruitful area for future work in non-clinical self-harming groups.

Borderline Personality Disorder is also known to share some affect regulation and impulse control features with attention-deficit/hyperactivity disorder (ADHD) and ADHD may be comorbid with BPD (Philipsen, 2006). Additionally, ADHD may be a risk factor for the development of BPD in adulthood (Philipsen et al., 2008). However, it is possible that attentional control problems may underlie both conditions, constituting the shared processes of each condition, and that the emergence of one disorder rather than the other, or one main disorder with ADHD co-morbidity, is driven by the selective constellation of personality, developmental and familial factors combined with attentional control problems. Future work might explore the potential shared contribution of executive/attentional control problems to personality disorders and co-morbid conditions.

A limitation of the current study was the use of self-report measures of attentional control although other work also indicates that ACS scores are associated with behavioral and neurophysiological indicators of executive control (e.g., Derryberry and Reed, 2002). It is possible that subjective reports differ about attentional control are not similar from objective indices of attentional control (Verwoerd et al., 2008). Consequently, our ongoing work is developing new experimental paradigms and using a comprehensive raft of standardized cognitive tests to investigate these assumptions and further tease apart the putative relationship between executive control and self-harm likelihood.

To summarize, present findings support the notion of a multi-componential executive system by demonstrating different patterns of relationship among attentional variables on likelihood of self-harm in those with BPD features. Of note, those high in BPD features showed high focusing scores indicating no impairment in this capacity as we anticipated, although flexibility and shifting scores were significantly lower in those with a self-harm history compared to non self-harmers. This finding seems to indicate that it is the content of attentional focusing rather than the process that may be pathological in those high in BPD features.

The high incidence of self-harm cases reported each year beyond psychiatric groups suggests a need for improved pathways to diagnosis and treatment for those who self-harm. Our data indicate that BPD features might play a role in mediating these behaviors and also that attentional control factors, as measured by our variables also contribute to self-harm likelihood. Overall, our findings indicate that personality and attentional control factors interact to determine self-harm likelihood whereby high attentional focusing and shifting abilities are protective when BPD features are low but high focusing may be a possible maintaining factor when BPD features are high.

## Conflict of interest statement

The authors declare that the research was conducted in the absence of any commercial or financial relationships that could be construed as a potential conflict of interest.

## Footnotes

^1^http://www.facebook.com/SHAwareness
